# Endothelial Response Boosted by Platelet Lysate: The Involvement of Calcium Toolkit

**DOI:** 10.3390/ijms21030808

**Published:** 2020-01-26

**Authors:** Simona Martinotti, Mauro Patrone, Valeria Balbo, Laura Mazzucco, Elia Ranzato

**Affiliations:** 1 DiSIT—Dipartimento di Scienze e Innovazione Tecnologica, University of Piemonte Orientale, Viale Teresa Michel 11, 15121 Alessandria, Italy; mauro.patrone@uniupo.it (M.P.); elia.ranzato@uniupo.it (E.R.); 2 DiSIT—Dipartimento di Scienze e Innovazione Tecnologica, University of Piemonte Orientale, piazza Sant’Eusebio 5, 13100 Vercelli, Italy; 3 Laboratorio Produzione Emocomponenti e Medicina Rigenerativa, SIMT—AO “SS Antonio e Biagio”, 15121 Alessandria, Italy; vbalbo@ospedale.al.it (V.B.); lmazzucco@ospedale.al.it (L.M.)

**Keywords:** cell calcium, endothelial cells, platelet lysate, ROS, wound repair

## Abstract

Wound repair is a dynamic process during which crucial signaling pathways are regulated by growth factors and cytokines released by several kinds of cells directly involved in the healing process. However, the limited applications and heterogeneous clinical results of single growth factors in wound healing encouraged the use of a mixture of bioactive molecules such as platelet derivatives for best results in wound repair. An interesting platelet derivative, obtained from blood samples, is platelet lysate (PL), which has shown potential clinical application. PL is obtained from freezing and thawing of platelet-enriched blood samples. Intracellular calcium (Ca^2+^) signals play a central role in the control of endothelial cell survival, proliferation, motility, and differentiation. We investigated the role of Ca^2+^ signaling in the PL-driven endothelial healing process. In our experiments, the functional significance of Ca^2+^ signaling machinery was highlighted performing the scratch wound assay in presence of different inhibitors or specific RNAi. We also pointed out that the PL-induced generation of intracellular ROS (reactive oxygen species) via NOX4 (NADPH oxidase 4) is necessary for the activation of TRPM2 and the resulting Ca^2+^ entry from the extracellular space. This is the first report of the mechanism of wound repair in an endothelial cell model boosted by the PL-induced regulation of [Ca^2+^]_i_.

## 1. Introduction

Wound healing is a well-orchestrated process strictly dependent on correct functions of several cell types, such as inflammatory cells, endothelial cells, fibroblasts, and keratinocyte cells, with the ultimate goal of restoring tissue integrity [1]. 

This process relies on a complex integration of some cellular signaling, requiring multiple growth factors and cytokines stimuli, with positive and negative effects on different wound phases [2].

Some of these factors are well characterized, but until now, few of them are available for clinical use due to their short half-life and excessive cost. In particular, the use of single growth factor is not effective in affecting wound repair, and the best solution should be to deliver a mixture of several growth factors in order to induce complete repair [3,4].

Platelets are important elements in wound repair and tissue regeneration processes, affecting cell recruitment and extracellular matrix remodeling, as well as blood vessel growth and angiogenesis.

Platelets are characterized by the presence of granules containing growth factors, cytokines, and chemokines that are able to stimulate cellular activities at wounded sites [5].

An increasing amount of research has shown the positive role of growth factors, released by platelets, if delivered in wound healing sites, stimulating the healing process in a physiological fashion [6].

An attractive platelet derivative, showing potential clinical application, is platelet lysate (PL). PL is obtained from repeated freezing and thawing of platelet-enriched blood samples [5]. This PL contains a mixture of growth factors and other molecules able to stimulate tissue regeneration. Human PL has been also been proposed as an alternative to classic fetal bovine serum (FBS) to avoid xenogenic immune reactions as well as viruses and prion transmissions [7].

We have previously demonstrated by in vitro scratch wound, cell migration, and microscope image analysis that PL activates the repair mechanisms in various types of injured endothelial cell layers [8].

Our observations suggested that the PL-induced wound healing is driven by a complex of effects, including both cell proliferation and migratory behavior. These data confirm previous findings about the chemotactic responses to platelet derivatives of glial cells [9], rat fetal cells [10], osteoblasts [11], keratinocytes [12], and primary fibroblasts [13].

Cytosolic free Ca^2+^ is essential for cell proliferation and motility [14]. Moreover, the role of intracellular Ca^2+^ in wound healing has been suggested by wound-induced Ca^2+^ waves observed in injured cells, as well as by PL-induced oscillatory Ca^2+^ signaling [15]. 

Studies carried out on endothelial cells have shown wound-induced Ca^2+^ waves [16], while intracellular Ca^2+^ signaling is essential to promote wound healing during endothelial repair [17].

An increase in intracellular Ca^2+^ concentration [Ca^2+^]_i_ is a proposed trigger in wound healing, and Ca^2+^ waves spread from injured areas leading to temporary Ca^2+^ increase. Moreover, a correlation between Ca^2+^ dynamics and wound repair has been reported in studies on several cell types, i.e., keratinocytes, fibroblasts, and endothelial cells, where PL has induced significant Ca^2+^ signals, and the use of Ca^2+^ chelator BAPTA-AM (1,2-Bis(2-aminophenoxy)ethane-*N,N,N′,N′-*tetraacetic acid tetrakis-acetoxymethyl ester) in experiments of scratch wound healing suppressed the healing effect of PL [8,11,12,18].

Intracellular Ca^2+^ signaling plays a central role in stimulating wound healing; however, the mechanistic information on injury-induced Ca^2+^ waves in vascular endothelium is still under investigation [17].

The aim of this study is to further characterize the PL-induced wound healing processes investigating the involvement of the Ca^2+^ toolkit in the PL-boosted regeneration. Disclosing the molecular nature of the pathway gating Ca^2+^ entry into PL-stimulated endothelium is a crucial challenge to employ Ca^2+^ signals for therapeutic purposes.

## 2. Results

### 2.1. PL Induces Tissue Regeneration in a Dose-Dependent Manner

We have earlier demonstrated that in endothelial cells, PL exposure induced variable increases of cell viability and proliferation, with maximum effects observed at 20% PL [19].

**Figure 1:**
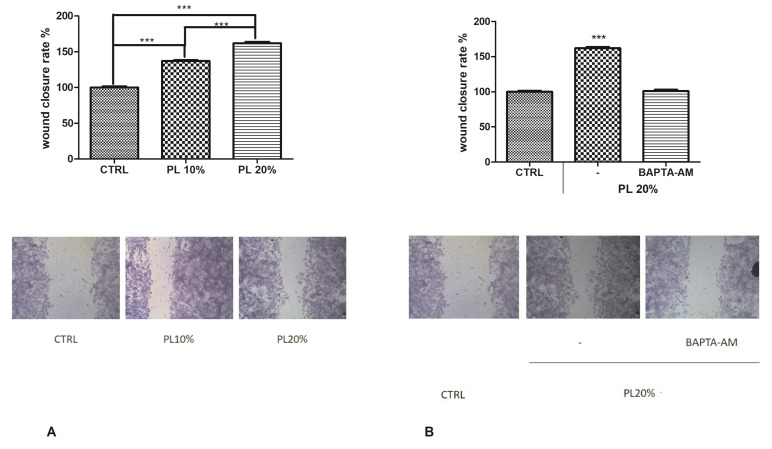
Platelet lysate (PL)-induced wound closure. (**A**) Upper panel: Effect of different concentrations of PL in scratch wound repair of bEND5 monolayers. PL was used; 10% and 20% *v/v*. CTRL are cells in control conditions, i.e., cells grown in DMEM with 10% FBS as described in the Materials and Methods section. Wound closure rate is expressed as the difference between wound width at 0 and 24 h. Data were recorded 24 h after scratch wound healing of cells exposed to PL. Bars represent mean ± S.E.M. of wound closure derived from two independent experiments, each with *n* = 20. Different asterisks on bars indicate statistical differences determined by one-way ANOVA with Tukey’s test (*p* < 0.05). Lower panel: Micrographs of scratch-wounded bEND5 monolayers incubated under control conditions (cells incubated in DMEM with 10% FBS) or in the presence of 10% and 20% PL and then stained with blue toluidine and observed 24 h after wounding. (**B**) Upper panel: Role of intracellular Ca^2+^ in PL-induced scratch wound repair of endothelial monolayers, in presence or not of 30 µM BAPTA-AM. Wound closure rate is expressed as the difference between wound width at 0 and 24 h. Data were recorded 24 h after scratch wound healing of cells exposed to PL. Bars represent mean ± S.E.M. of wound closure inhibition deriving from two independent experiments, each with *n* = 20. Asterisks on bars indicate statistical differences determined by one-way ANOVA with Tukey’s test (*p* < 0.001). Lower panel: Micrographs of scratch-wounded bEND5 monolayers incubated under control conditions (as described above) or in the presence of PL and BAPTA-AM and then stained with blue toluidine and observed 24 h after wounding.

To disclose the functional role of PL in tissue regeneration, we performed the scratch wound assay on bEND5 cells, in presence of 10% or 20% *v/v* PL (Figure 1A), confirming that 20% PL was the most effective concentration also in inducing wound closure.

Then, to demonstrate the involvement of Ca^2+^ signaling in the PL-induced wound closure mechanism, we repeated the scratch wound assay in presence or not of BAPTA-AM. We observed that this inhibitor significantly reduced the wound healing rate, highlighting the importance of extracellular Ca^2+^ entry (Figure 1B,C).

### 2.2. PL Induces Ca^2+^ Signals in a Dose-Dependent Manner

Based on the previously observed fundamental role of Ca^2+^ in the wound closure, we investigated whether and how PL determines variations in [Ca^2+^]_i_. Therefore, we assessed intracellular Ca^2+^ variations, by using time-lapse confocal microscopy imaging of bEND5 cells loaded with the fluorescent Ca^2+^ probe Fluo-3/AM. 

**Figure 2:**
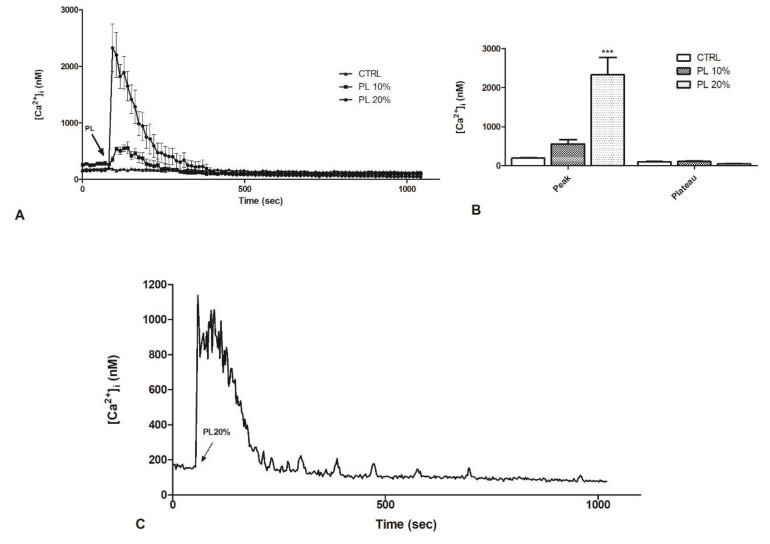
PL induces a dose-dependent increase in intracellular Ca^2+^ concentration in bEND5 cells. (**A**) [Ca^2+^]_i_ variations recorded at 10 s intervals, showing no variations in control conditions (CTRL, i.e., cells incubated in confocal microscopy loading buffer, as described in Materials and Methods section), and distinct patterns of Ca^2+^ signaling after exposure to 10% and 20% PL. Data are means ± s.e.m. of [Ca^2+^]_i_ traces recorded in different cells. Number of cells: CTRL: 20 cells from 2 experiments; 10% PL: 40 cells from 3 experiments; 20% PL: 40 cells from 3 experiments. (**B**) Mean ± s.e.m. of the peak Ca^2+^ response induced by treatment with 10% or 20% PL. Number of cells: CTRL: 20 cells from 2 experiments; 10% PL: 40 cells from 3 experiments; 20% PL: 40 cells from 3 experiments. Asterisks on bars indicate statistical differences determined by two-way ANOVA with Bonferroni’s correction (*p* < 0.001). **C**. [Ca^2+^]_i_ variations recorded at 1 s intervals induced by 20% PL. Data are means ± s.e.m. of [Ca^2+^]_i_ traces recorded in 40 different cells.

The analysis of confocal imaging of Fluo-3/AM loaded bEND5 cells exposed to 20% PL revealed a single, large [Ca^2+^]_i_ spike. After PL exposure, the spike started immediately reaching the peak and returned to basal line in approximately 400 s (Figure 2A,B). Ten percent PL induced only a smaller peak, and the [Ca^2+^]_i_ returned to the basal line in 250 s (Figure 2A,B). These observations showed that [Ca^2+^]i, sampled in bEND5 cells at 10 s intervals, did not undergo any spontaneous oscillations in control conditions (Figure 2A).

We decided to investigate if the increase in [Ca^2+^]_i_ had fluctuations over time; hence, we repeated the analysis by confocal imaging of Fluo-3/AM loaded bEND5 cells exposed to 20% PL at 1 s intervals. When the peak was reached, oscillations were observed for about 100 s. Subsequently, there was a decrease until reaching the basal level. However, before the signal stabilization, small oscillations could be observed that gradually decreased in intensity and frequency (Figure 2C).

### 2.3. Contribution of Extracellular Ca^2+^ to Cytosolic Increase

**Figure 3:**
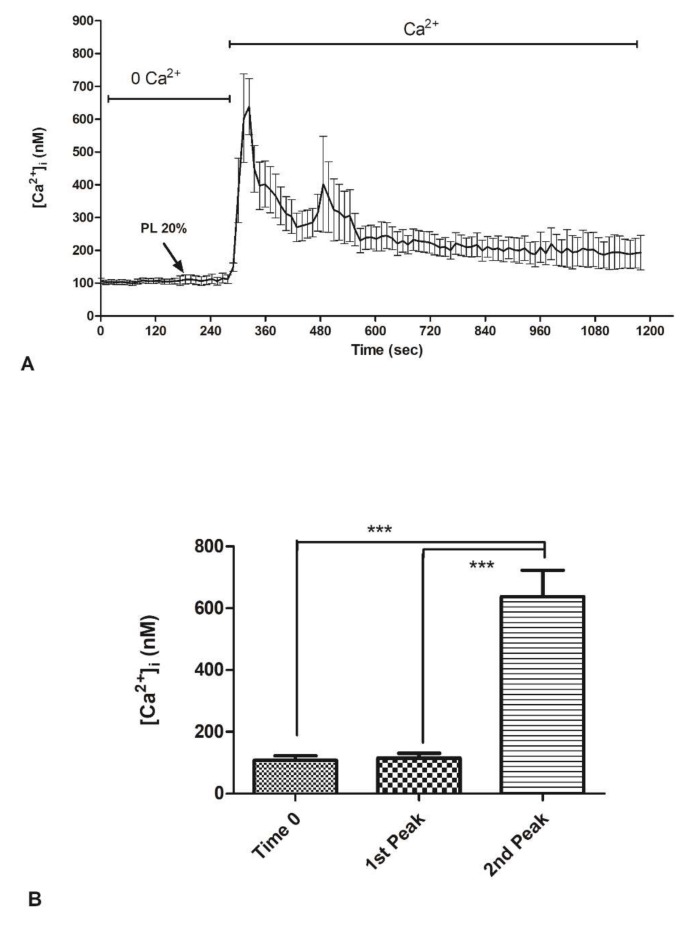
PL-induced Ca^2+^ entry from the extracellular space in bEND5 cells. (**A**) To monitor exclusively intracellular Ca^2+^ mobilization, bEND5 cells were stimulated with 20% PL in the absence of external Ca^2+^ (0Ca^2+^). When Ca^2+^ has been added back to the medium, there was [Ca^2+^]_i_ increase. (**B**) Mean ± s.e.m. of the Ca^2+^ response at time zero and to 20% PL recorded after PL addition (first peak) and at the peak after Ca^2+^ supplement in extracellular space (second peak). Data are means ± s.e.m. of [Ca^2+^]_i_ measured by confocal imaging of 40 cells from 3 experiments Different asterisks on bars indicate statistical differences by one-way ANOVA according to Tukey’s test (*p* < 0.001).

In order to assess which Ca^2+^ sources underlie these Ca^2+^ signals, 20% PL was applied in the absence of external Ca^2+^ (0Ca^2+^), after which Ca^2+^ was reintroduced in the continued presence of the agonist. PL did not cause any increase in [Ca^2+^]_i_ under 0Ca^2+^ conditions, which reflects the fundamental role of extracellular Ca^2+^ (Figure 3A,B). The subsequent restitution of Ca^2+^ to the medium induced an elevation [Ca^2+^]_i_ which depends on extracellular Ca^2+^ entry (Figure 3A,B). 

We have knowledge that transient receptor potential cation channel, subfamily M, member 2 (TRPM2) is highly expressed in endothelial cells where it mediates redox-activated Ca^2+^ entry [20]. Hence, based on our data defining ROS production after PL treatment, to better define the Ca^2+^ entry from the extracellular environment under PL exposure, we focused on the role of TRPM2 channel. 

**Figure 4:**
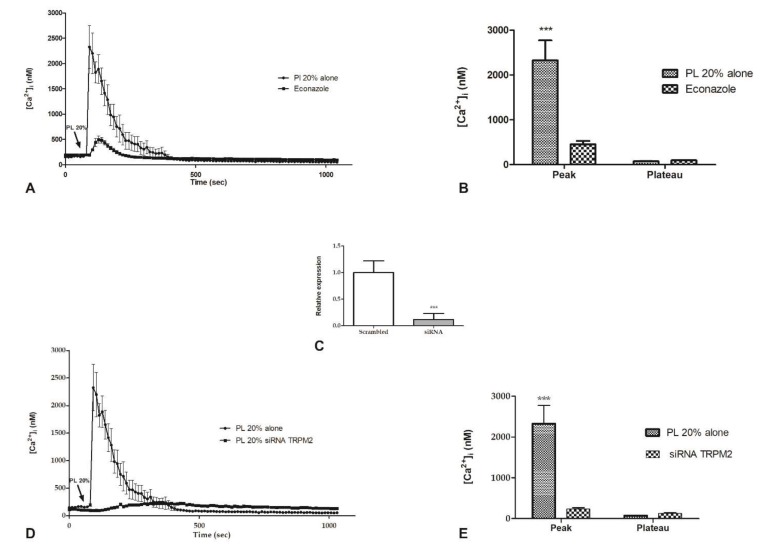
The inhibition of TRPM2 channel reduced PL-induced Ca^2+^ entry. (**A**) PL-induced Ca^2+^ entry was dramatically reduced in the presence of 10 µM Econazole (30 min preincubation). PL was added at 20% *v/v*. Data are means ± s.e.m. of [Ca^2+^]_i_ traces recorded in different cells. Number of cells: PL alone: 40 cells from 3 experiments; PL + Econazole 10 µM: 50 cells from 3 experiments. (**B**) Mean ± s.e.m. of the Ca^2+^ response to 20% *v/v* PL recorded at the peak and at the plateau under the designated treatments. Data are means ± s.e.m. of [Ca^2+^]_i_ measured by confocal imaging at peak maxima. Number of cells: PL alone: 40 cells from 3 experiments; PL + Econazole 10 µM: 50 cells from 3 experiments. Asterisks on bars indicate statistical differences determined by two-way ANOVA with Bonferroni’s correction (*p* < 0.001). (**C**) Expression of TRPM2 gene in bEND5 cells after RNAi. The mRNA quantity of TRPM2 was determined by qRT-PCR and is represented as mean relative expression ± SD (*n* = 3, * *p* < 0.001, *t*-test). (**D**) PL-induced Ca^2+^ entry was completely abrogated in cells transfected with RNAi targeting TRPM2. PL was added at 20% *v/v*. Data are means ± s.e.m. of [Ca^2+^]_i_ traces recorded in different cells. Number of cells: PL alone: 30 cells from 3 experiments; PL after RNAi for TRPM2: 50 cells from 3 experiments. (**E**) Mean ± S.E.M. of the Ca^2+^ response to 20% *v/v* PL recorded at the peak and at the plateau under the designated treatments. Data are means ± s.e.m. of [Ca^2+^]_i_ measured by confocal imaging at peak maxima. Number of cells: PL alone: 30 cells from 3 experiments; PL after RNAi for TRPM2: 50 cells from 3 experiments. Asterisks on bars indicate statistical differences determined by two-way ANOVA with Bonferroni’s correction (*p* < 0.001).

Confocal imaging showed that bEND5 cell preincubation (30 min) with 10 µM econazole, a TRPM2 inhibitor [21], was able to reduce the [Ca^2+^]_i_ increase caused by 20% PL exposure (Figure 4A), determining a significantly reduction of the amplitude of Ca^2+^-peak in response to PL (Figure 4B). The depletion of TRPM2 channel by RNAi (Figure 4C) determines the complete abrogation of the peak in [Ca^2+^]_i_ increase after 20% PL exposure (Figure 4D,E).

### 2.4. Intracellular ROS Generation

**Figure 5:**
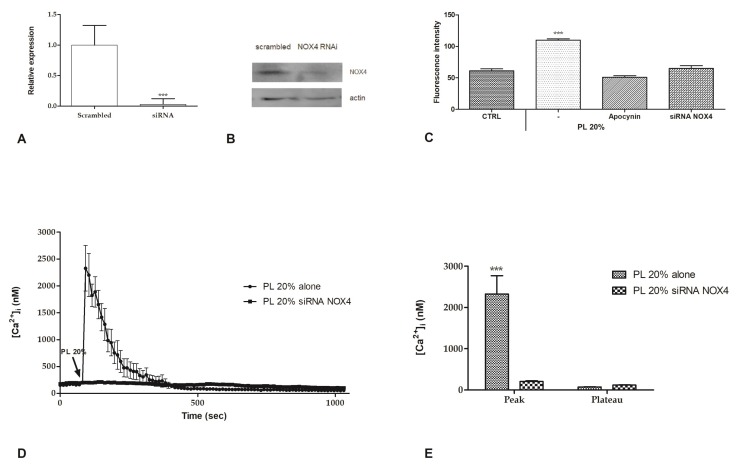
PL-induced ROS production and their involvement in Ca^2+^ entry. (**A**) Expression of NOX4 gene in bEND5 cells after RNAi. The mRNA quantity of NOX4 was determined by qRT-PCR and is represented as mean relative expression ± SD (*n* = 3, * *p* < 0.001, *t*-test). (**B**). NOX4 protein expression in scrambled cells or after NOX4 RNAi. Blots representative of three were shown. Lanes were loaded with 25 μg of proteins, then probed with anti-NOX4 antibody, and managed as described in the Materials and Methods. The same blots were stripped and re-probed with anti-actin antibody. (**C**) ROS production in DHR-123 loaded cells recorded at 120 s after 20% PL exposure. CTRL are cells incubated in confocal microscopy loading buffer, as described in Materials and Methods section. Bars represent mean ± s.e.m. of ROS production deriving from two independent experiments, each with *n* = 20. Asterisks on bars indicate statistical differences determined by one-way ANOVA with Dunnet’s test (*p* < 0.001). (**D**) PL-induced Ca^2+^ entry was completely abrogated after RNAi for NOX4. PL was added at 20% *v/v*. Data are means ± s.e.m. of [Ca^2+^]_i_ traces recorded in different cells. Number of cells: PL alone: 40 cells from 3 experiments; PL after RNAi for NOX4: 50 cells from 3 experiments. **(E)**. Mean ± s.e.m. of the Ca^2+^ response to 20% *v/v* PL recorded at the peak and at the plateau under the designated treatments. Data are means ± s.e.m. of [Ca^2+^]_i_ measured by confocal imaging at peak maxima. Number of cells: PL alone: 40 cells from 3 experiments; PL after RNAi for NOX4: 50 cells from 3 experiments. Asterisks on bars indicate statistical differences determined by two-way ANOVA with Bonferroni’s correction (*p* < 0.001).

It is commonly known that some growth factors (GFs), such as PDGF [22] and VEGF [23], are able to stimulate the generation of intracellular ROS by activation of NAPH oxidases complex [24]. To determine if also treatment with PL is able to induce this pathway, we assessed the generation of intracellular ROS after addition of 20% PL by using dihydrorhodamine-123 (DHR-123). Intracellular levels of ROS were revealed using the fluorescent dye DHR-123, which is converted to fluorescent rhodamine-123 upon reaction with ROS. We observed that PL induces the increase of intracellular ROS. Data were confirmed by the absence of this rise in presence of 50 µM apocynin, a NOX (NADPH oxidase) inhibitor, or siRNA for NOX4 (Figure 5A–C). 

Confocal imaging of bEND5 cells treated with RNAi for NOX4 showed that the [Ca^2+^]_i_ increase caused by 20% PL exposure was completely abrogated (Figure 5D,E). 

### 2.5. Contribution of Intracellular Stores to Cytosolic Ca^2+^ Increase

It is well known that GFs binding to their specific receptors, belonging to G-protein-coupled receptors (GPCRs) and tyrosine kinase receptors (TKRs) families, determine an increase in [Ca^2+^]_i_, through the engagement of phospholipase C (PLC) that leads to PIP_2_ hydrolysis in InsP_3_ and DAG. 

For this reason, we focused on the contribution of the ER in the variation of [Ca^2+^]_i_ in response to PL. For this purpose, we utilized a panel of inhibitors, i.e., thapsigargin, 2-APB, U73122, and caffeine.

**Figure 6:**
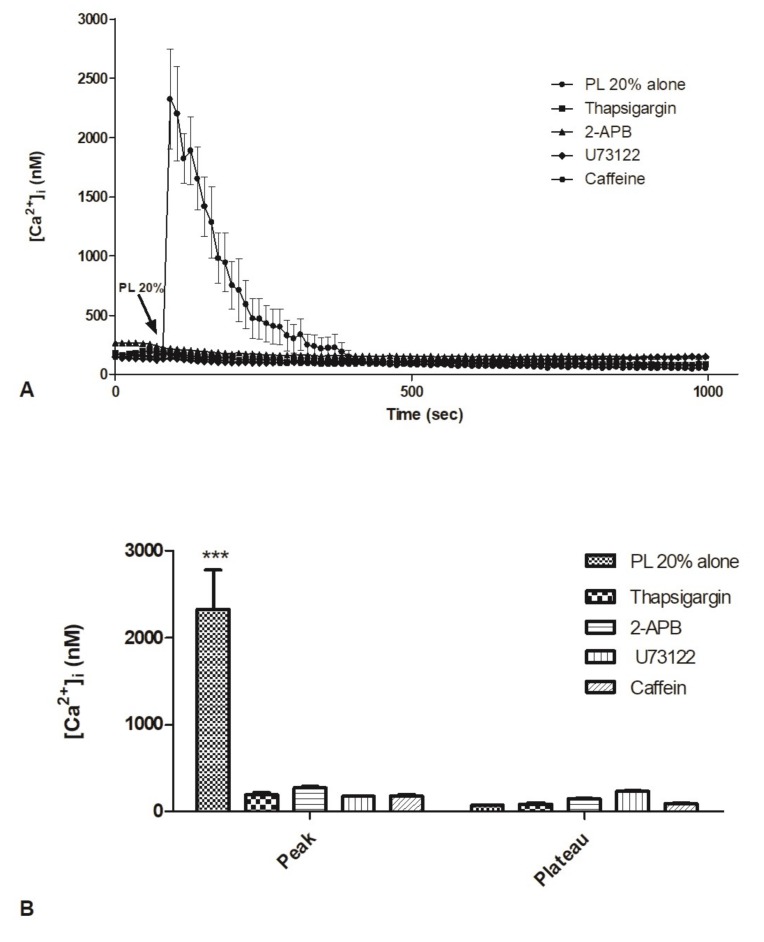
Contribution of intracellular stores to cytosolic Ca^2+^ increase. **A**. PL-induced Ca^2+^ release was dramatically reduced in the presence of thapsigargin, 2-APB, U73122, caffeine (30 min pre-incubation for each drug). PL was added at 20%. Data are means ± s.e.m. of [Ca^2+^]i traces recorded in different cells. Number of cells: PL 20% alone: 20 cells from 3 experiments; PL 20% + thapsigargin 5 µM: 40 cells from 3 experiments; PL 20% + 2-APB 50 µM: 45 cells from 3 experiments; PL 20% + U73122 10 µM: 50 cells from 3 experiments; PL 20% + caffeine 10 mM. **B**. Mean ± s.e.m. of the Ca^2+^ response to 20% PL recorded at the peak and at the plateau under the designated treatments. Data are means ± s.e.m. of [Ca^2+^]_i_ measured by confocal imaging at peak maxima. Number of cells: PL 20% alone: 20 cells from 3 experiments; PL 20% + thapsigargin 5 µM: 40 cells from 3 experiments; PL 20% + 2-APB 50 µM: 45 cells from 3 experiments; PL 20% + U73122 10 µM: 50 cells from 3 experiments; PL 20% + caffeine 10 mM. Asterisks on bars indicate statistical differences determined by two-way ANOVA with Bonferroni’s correction (*p* < 0.001).

Confocal imaging showed that bEND5 cell incubation with thapsigargin (5 µM, 30 min), a non-competitive inhibitor of the sarco/endoplasmic reticulum Ca^2+^ ATPase, was able to completely reduce the [Ca^2+^]_i_ increase triggered by PL exposure (Figure 6A,B). 

2-APB (50 µM, 30 min preincubation) and caffeine (10 mM, 30 min preincubation), two different blockers of InsP_3_R [25,26], induced the same effects, reducing the amplitude of the peak of the Ca^2+^ response to PL (Figure 6A,B). 

Likewise, U73122 (10 µM, 30 min preincubation), a PLC inhibitor [27], determined a significant decrease of PL-induced rise in [Ca^2+^]_i_ (Figure 6A,B). 

Cell incubation with vehicle DMSO used alone is ineffective on Ca^2+^.

### 2.6. Involvement of Store-Operated Ca^2+^ Entry

**Figure 7:**
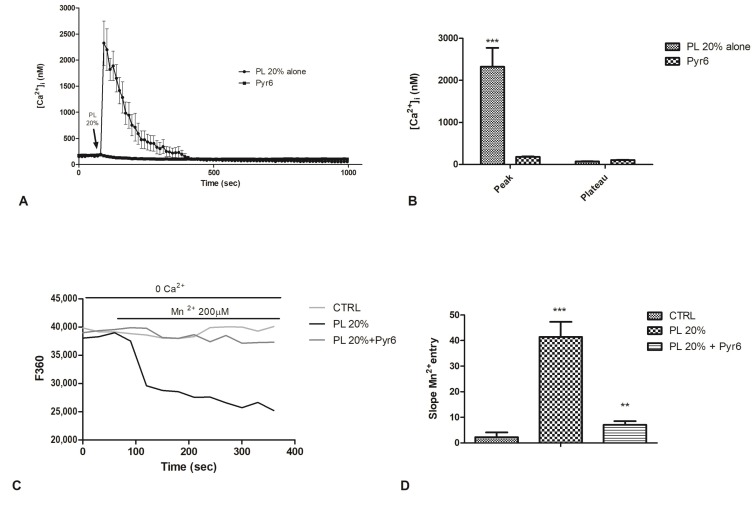
The inhibition of Orai1 channel abolished PL-induced Ca^2+^ entry. **A.** PL-induced Ca^2+^ entry was completely abolished in the presence of 1 µM Pyr6 (30 min preincubation). PL was added at 20% *v/v*. Data are means ± s.e.m. of [Ca^2+^]_i_ traces recorded in different cells. Number of cells: PL alone: 40 cells from 3 experiments; PL + Pyr6 1 µM: 50 cells from 3 experiments. **B**. Mean ± s.e.m. of the Ca^2+^ response to 20% PL recorded at the peak and at the plateau under the designated treatments. Data are means ± s.e.m. of [Ca^2+^]_i_ measured by confocal imaging at peak maxima. Number of cells: PL alone: 40 cells from 3 experiments; PL + Pyr6 1 µM: 50 cells from 3 experiments. Asterisks on bars indicate statistical differences determined by two-way ANOVA with Bonferroni’s correction (*p* < 0.001). **C.** Using the Mn^2+^-quenching technique, the resting Ca^2+^ entry in bEND5 cells was evaluated. First, the extracellular medium was replaced with a 0Ca^2+^ solution and then, to cause an immediate decay in Fura-2 fluorescence, 200 µM Mn^2+^ was added. PL 20% treatment allowed an evident decay of fluorescence, while pre-incubating the cells with Pyr6 strongly prevented this decay. CTRL indicates cells incubated in 0Ca^2+^ solution. **D.** The quenching rate of Fura-2 fluorescence induced by Mn^2+^ addition was calculated as the slope of a linear regression. Different asterisks on bars indicate statistical differences (*** *p* < 0.001; ** *p* < 0.005).

The Ca^2+^ pool reduction in the ER, following to InsP_3_-dependent Ca^2+^ release, led to the activation of store-operated Ca^2+^ entry (SOCE) [28,29]. Therefore, we investigated the involvement of SOCE in PL-stimulated cells. Pyr6 (1µM, 30 min), a selective inhibitor of Orai1 channels [30,31], caused a complete decrease of peak phase of PL-boosted elevation in [Ca^2+^]_i_ (Figure 7A,B). 

As recently discussed, in endothelial cells, resting conditions determine the partial activation of SOCE, leading to a control of ER Ca^2+^ refilling in bEND5 cells [32]. For this reason, to corroborate Pyr6 data, we then utilized the Mn^2+^-quenching technique, an established tool to monitor agonist-induced Ca^2+^ entry in vascular endothelial cells to assess whether 1) SOCE is active in un-stimulated cells and 2) PL stimulation may affect the rate of SOCE activation in bEND5 cells [33,34]. 

It is possible to use Mn^2+^ as a consistent surrogate of Ca^2+^ because it flows through most Ca^2+^-permeable channels. Considering the unidirectional influx of Mn^2+^, quenching of Fura-2 fluorescence, may be used as an index for stimulated cation entry. 

Figure 7C,D shows that a Ca^2+^-free extracellular solution (0Ca^2+^ 0EGTA), containing 200 µM Mn^2+^, enhanced the quenching of Fura-2 fluorescence, suggesting a constitutive Ca^2+^ entry pathway in bEND5 cells [32]. To assess whether PL stimulates SOCE in bEND5 cells, PL 20% was added once the rate of basal quenching had been established, and in this condition, we observed an increase in the slope of the quenching curve (Figure 7C,D). The pharmacological blockade of SOCE with Pyr6 prevented PL-induced divalent cation entry (Figure 7C,D). 

### 2.7. Functional Role of PL-Induced Tissue Regeneration 

**Figure 8:**
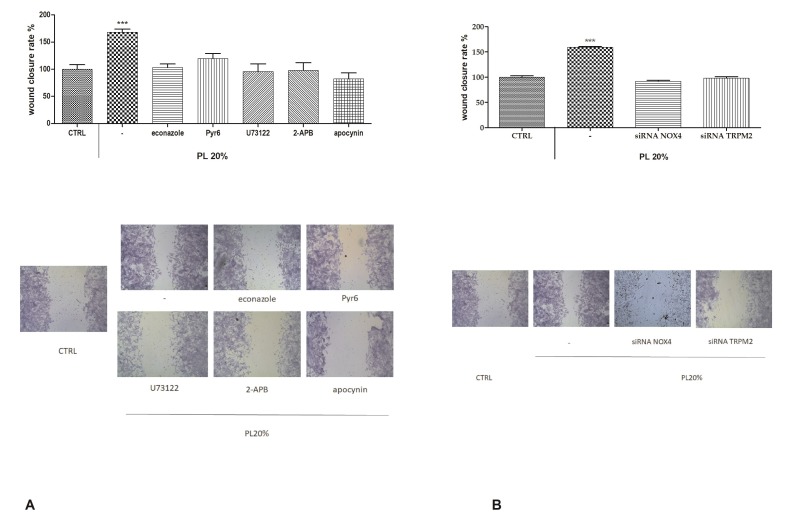
Role of intracellular ROS and Ca^2+^ in PL-induced wound closure. **A.** Upper panel: Effect of different inhibitors on PL-induced scratch wound repair of endothelial monolayers. Each inhibitor was used according to the procedures, which were previously shown to inhibit PL extracellular Ca^2+^ entry. CTRL are cells in control conditions, i.e., cells grown in DMEM with 10% FBS as described in the Materials and Methods section. Wound closure rate is expressed as the difference between wound width at 0 and 24 h. Data were recorded 24 h after scratch wound healing of cells exposed to PL. Bars represent mean ± s.e.m. of wound closure inhibition deriving from two independent experiments, each with *n* = 20. Asterisks on bars indicate statistical differences determined by two-way ANOVA with Bonferroni’s correction (*p* < 0.001). **Lower panel:** Micrographs of scratch-wounded bEND5 monolayers incubated under control conditions or in the presence of PL with different inhibitors. **B.** Upper panel: Effect of RNAi for NOX4 and TRPM2 on PL-induced scratch wound repair of endothelial monolayers. Wound closure rate is expressed as the difference between wound width at 0 and 24 h. Data were recorded 24 h after scratch wound healing of cells exposed to PL. Bars represent mean ± s.e.m. of wound closure inhibition deriving from two independent experiments, each with *n* = 20. Asterisks on bars indicate statistical differences determined by two-way ANOVA with Bonferroni’s correction (*p* < 0.001). Micrographs of scratch-wounded bEND5 monolayers incubated under control conditions (scrambled cells) or in the presence of PL and RNAi (siRNA) for NOX4 and TRPM2 and then stained with blue toluidine and observed 24 h after wounding.

To further confirm our observations, we repeated the scratch wound assay under different conditions of inhibition, i.e., econazole and siRNA for TRPM2 channel, Pyr6, U73122, 2-APB, and apocynin and siRNA for NOX4. In presence of all of these, we could observe a significant reduction of the wound closure rate (Figure 8).

## 3. Discussion

Wound repair is a dynamic process during which crucial signaling pathways are regulated by growth factors and cytokines released by several kinds of cells directly involved in the healing process.

However, the limited applications and heterogeneous clinical results [35] of single growth factors in wound healing encouraged the use of a mixture of bioactive molecules, such as platelet derivatives, for best results in wound repair.

PL, obtained by repeated freezing/thawing of blood platelets, showed to release more growth factors compared to platelet-rich plasma [36]. Moreover, to standardize the PL product, we obtained it from a pool of healthy donors, to minimize individual variability. We have previously validated that PDGF-AB amounts, one of most abundant growth factors in PL, resulted in the same order of size of previous studies on PL [37].

We have already observed as cell viability and proliferation, cell migration induction, and scratch wound assay, revealed that 20% PL promotes cellular activity, accelerating in vitro wound healing in various cell types [8,11,12,13,38,39], including endothelial cells [8]. 

The integrity of an endothelial monolayer may be compromised by either disturbed blood flow or pathological conditions [17]. Moreover, the prolonged and/or repeated exposure of the endothelial monolayer to well-known cardiovascular risk factors (i.e., hypertension, hyperlipidemia, hypercholesterolemia, smoking, and ageing) has a dramatic impact on vascular endothelium [17]. Vascular injury causes endothelial cells to lose integrity, progress to senescence, undergo apoptosis, and ultimately slough off the vessel wall into the bloodstream. Endothelial integrity may then be disturbed by several surgical procedures aiming at restoring blood flow. It is therefore not surprising that novel approaches are crucial for clinicians, to achieve the early restoration of a fully competent endothelium in order to gain more effective long-term results after vascular regenerative surgery [17].

Intracellular Ca^2+^ signals play a central role for control endothelial survival, proliferation, motility, and differentiation [29]. Ca^2+^ entry from the extracellular environment usually triggers long-lasting cytosolic Ca^2+^ signals of smaller amplitude compared to the Ca^2+^ release-related spikes. The best-characterized endothelial Ca^2+^ channels are voltage independent, even if voltage-dependent Ca^2+^ fluxes have been described in primary endothelial cells [29]. Moreover, store-operated calcium entry (SOCE) is of relevance to sustain endothelial proliferation, migration, gene expression, and NO synthesis [40]. Likewise, it is well known that Ca^2+^ plays a crucial role in guiding the tissue regeneration mechanism [41]. 

In this study, we investigated the role of Ca^2+^ signaling in the PL-driven endothelial healing process. Immediately after 20% PL addition, it was possible to detect a huge increase in [Ca^2+^]_i_ level, characterized by a 100 sec oscillations peak phase, followed by a decrease and stabilization phase also featured by the presence of less intense and less frequent oscillations. This increase was completely prevented by using different inhibitors. 

We observed that in absence of Ca^2+^ in the extracellular space, the PL-induced signaling cascade was not triggered, but reestablishment of basal [Ca^2+^]_e_ generates again a Ca^2+^ response observable by the presence of two spikes. These findings demonstrate that the onset of the Ca^2+^ increase after PL addiction depend on Ca^2+^ influx from extracellular space that is required to sustain the intracellular Ca^2+^ signal.

In this Ca^2+^ influx plays a pivotal role the TRPM2 channel that is well known to be activated by intracellular ROS. PL, due to its growth factors, is able to generate ROS in the intracellular space. GFs, after binding to their specific receptors, activate the NADPH oxidase complex that led to an increase of intracellular ROS, as we observed with DHR-123 fluorescent probe.

The existence of the link between PL, TRPM2, and intracellular ROS was confirmed by pharmacological inhibition through apocynin and by RNAi for NOX4, which lead to a reduction of Ca^2+^ entry. 

Further, Ca^2+^ release from the ER is fundamental to sustain the PL-induced cytosolic Ca^2+^ increase. Our data suggest that depletion of Ca^2+^ in the ER after pretreatment with thapsigargin determined the complete abrogation of cytosolic Ca^2+^ rise. 

Moreover, PLC/InsP_3_-mediated Ca^2+^ release showed a pivotal role in PL-induced endothelial tissue regeneration: the inhibition of PLCγ using U73122 [27] and of InsP_3_R by using 50 µM 2-APB [42] or 10 mM caffeine [43] determined a complete abolition of the PL-induced intracellular Ca^2+^ increase. 

The activation of SOCE during PL-induced Ca^2+^ signaling was determined inhibiting Orai1 channel with Pyr6: in this condition, we could not observe any variations in [Ca^2+^]_i_. Such observation was confirmed by using the Mn^2+^-quenching technique [32] that revealed a Ca^2+^ influx pathway compatible with that of SOCE as a result of PL treatment in bEND5 cells. The slope of this Ca^2+^ entry was almost completely nullified in the presence of Pyr6. 

Overall, these data suggest a role of intracellular Ca^2+^ stores in the PL-induced increase of cytosolic Ca^2+^: in particular, we observed an involvement of InsP_3_ and InsP_3_R in the transducing pathway of PL-induced Ca^2+^ spike; hence, we could speculate a role of PLC, through InsP_3_-sensitive channels. Moreover, the correct replenishment of the ER by SERCA pump and SOCE activation are equally fundamental. 

Intracellular Ca^2+^ is essential for cell proliferation and motility [44], and its role in wound healing has been repetitively described, also in endothelial cells [45], while intracellular Ca^2+^ rises have been found to promote cell growth and movement throughout endothelial repair [46]. 

We have already described a correlation between Ca^2+^ dynamics and wound repair in studies on skin cells, such as keratinocytes and fibroblasts, where PL induced evidentCa^2+^ signals, and BAPTA-AM abrogated the PL effect on wound healing [12,38]. 

In our experiments, the functional significance of a Ca^2+^ toolkit was highlighted when performing the scratch wound assay in presence of different inhibitors or specific RNAi. In every condition, the wound closure rate was heavily reduced compared to the one given by PL. We also pointed out that the generation of intracellular ROS via NOX4, boosted by PL induction, is necessary for the activation of TRPM2 and the resulting Ca^2+^ entry from the extracellular space.

**Figure 9:**
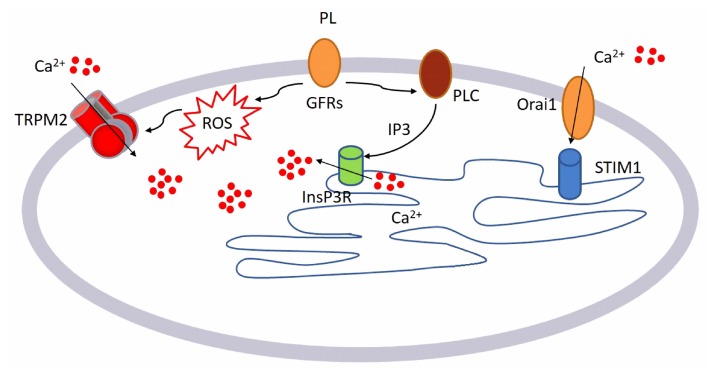
Diagram depicting the mechanism of action of PL on bEND5 endothelial cells, as characterized in the study.

This is the first report investigating the PL-induced regulation of [Ca^2+^]_i_ boosting wound repair in endothelial cell model. We can summarize our results as described in the following model (see Figure 9):

- GFs contained in PL bind to their specific receptors
- This binding leads to the activation of two different pathways:
  ○ Generation of intracellular ROS via NOX4
  ○ Activation of PLCγ, leading to the production of InsP_3_
- Intracellular ROS activates TRPM2 channel and consequently Ca^2+^ entry from the extracellular space.

InsP_3_ determines the release of Ca^2+^ from the intracellular store, activating the SOCE that operates to reload the intracellular stores sustaining the PL Ca^2+^ response.

Data showed that PL could trigger intracellular Ca^2+^ changes. PL induced, via intracellular ROS generation, the activation of TRPM2 that in turn determined Ca^2+^ entry from the extracellular space. Pharmacological and genetic (siRNA) inhibition of TRPM2 inhibited the Ca^2+^ response to PL.

At the same time, GFs binding to their receptors induces PLC activation and consequently the release of Ca^2+^ from the ER through InsP_3_R, confirmed by the pharmacological inhibition of the PLC/InsP_3_ cascade. Therefore, the pharmaceutical inhibition of Orai1 also diminished the Ca^2+^ response to PL, suggesting the involvement of SOCE in sustaining of response to PL. 

Moreover, our findings can also represent an important knowledge for the use of PL as an alternative for ex-vivo expansion of endothelial cells before clinical administration, avoiding issues related to the use of fetal bovine serum. 

## 4. Materials and Methods 

### 4.1. Cell Culture and Reagents

All reagents were from Sigma-Aldrich, unless otherwise indicated. 

The bEND5 cell line (American Type Culture Collection, Manassas, VA, USA) is an immortalized mouse cell line from brain capillary endothelial cells. Cells were grown at 37 °C, 5% CO_2_ in DMEM (high glucose, 4.5 g/L), supplemented with 10% FBS, L-glutamine (200 mM), 100 U/mL penicillin, and 100 mg/mL streptomycin [8]. 

### 4.2. Platelet Lysate (PL) Preparation

Platelet concentrates were obtained following a standard clinical method. A platelet-enriched fraction was purified from platelet concentrate obtained according to standard procedures for the preparation of blood components. To obtain PL, the platelets concentrate, at the density of 1 × 10^9^ cell/mL, was washed three times in Ham’s F10 medium, 0.3% ethylenediamine tetraacetic acid (EDTA) to remove possible traces of plasma factors. To complete PL preparation, the concentrated washed platelets were then subjected to thermal shock: frozen (−80 °C) and thawed (37 °C). Platelet bodies and debris were eliminated by centrifugation and the supernatant was stored in aliquots at −80 °C until use [8,12,38,47].

### 4.3. Scratch Wound Test

Scratch wounds were made in confluent monolayers by using a sterile 0.1–10 µL pipette tip. After washing away suspended cells, cultures were refed with medium in the presence of PL for 24 h. After cell exposures, cells were fixed for 30 min in 3.7% formaldehyde prepared in PBS for 30 min, and then stained for 30 min at room temperature with 0.1% toluidine blue for 30 min. The wound space width was measured at wounding and at the end of treatments, using an inverted microscope (Leica Microsystems, Wetzlar, Germany) equipped with a digital camera. Digitized pictures of wounds were analyzed using the NIH Image J software. Wound closure was determined as the difference between wound width at 0 h and at 24 h.

### 4.4. Western Blotting

Cells were lysed in RIPA buffer (supplemented with a protease and phosphatase inhibitor cocktail) and homogenates were solubilized in Laemmli buffer. Amounts of 25 μg of proteins were loaded on gel, subjected to SDS-PAGE (12% gel), and then transferred to a nitrocellulose membrane, using a Bio-Rad Mini Trans Blot electrophoretic transfer unit (Bio-Rad Laboratories, Hercules, CA, USA). Membranes were blocked with 5% nonfat dry milk in PBS and then probed at 4 °C overnight, with a primary antibody against NADPH oxidase (NOX)–4 (dilution, 1:500; AbCam, Cambridge, UK). Membranes were then washed three times with PBS-0.05% Tween-20 to remove unbound antibodies and further incubated with appropriate horseradish peroxidase-conjugated secondary antibodies (dilution, 1:1000; Bethyl Laboratories, Montgomery, TX, USA). Membranes were developed using an enhanced chemiluminescence kit (Millipore, Billerica, MA, USA) according to the manufacturer’s protocol and digitized with Quantity One Image Software (ChemiDoc XRS; Bio-Rad). Equal loadings were confirmed with an anti-actin antibody (Santa Cruz Biotechnology, Paso Robles, CA, USA).

### 4.5. Measurements of Intracellular ROS

Intracellular levels of ROS were evaluated using dihydrorhodamine (DHR) 123, a fluorescent dye precursor, which is converted to fluorescent rhodamine 123 upon reaction with ROS. 

Cells were plated on glass-base dishes (Iwaki Glass, Inc., Tokyo, Japan), allowed to settle overnight, and loaded for 30 min at room temperature in the dark with DHR-123 (30 µM) in a loading buffer consisting of (mM) 10 glucose, 10 Hepes, 140 NaCl, 2 CaCl_2_, 1 MgCl_2_, and 5 KCl, pH 7.4. After probe loading and washing, cells were observed through confocal time-lapse analysis, using a Zeiss LSM 510 confocal system interfaced with a Zeiss Axiovert 100 M microscope (Carl Zeiss Inc., Oberkochen, Germany). 

Excitation was acquired by the 488 nm line of an Ar laser, and emission was collected using a 505–550 bandpass filter. ROS production data were expressed as fluorescence arbitrary units [48].

### 4.6. Measurements of Free Cytosolic Ca^2+^ Concentration ([Ca^2+^]_i_)

Cells were plated on glass-base dishes (Iwaki Glass, Inc., Tokyo, Japan), allowed to settle overnight, and then loaded with Fluo-3/AM (20 mM), a cell-permeant, fluorescent calcium probe in the dark for 30 min at 37 °C. The loading buffer consisted of (mM) 140 NaCl, 10 glucose, 10 HEPES pH 7.4, 2 CaCl_2_, 5 KCl, and 1 MgCl_2_. For Ca^2+^-free experiments, the ion was omitted from the loading buffer [38,49,50].

After probe loading and washing, cells were analyzed through confocal time-lapse analysis, using a Zeiss LSM 510 confocal system interfaced with a Zeiss Axiovert 100 M microscope (Carl Zeiss Inc., Oberkochen, Germany). 

Excitation was realized by the 488 nm line of an Ar laser, and emission was collected using a 505–550 bandpass filter. 

Several cells were viewed together through a 20× Plan-Neofluar Zeiss objective (0.5 NA). Fluo-3/AM fluorescence was evaluated in digitized images as the average value over defined contours of individual cells. Fluo-3 calibration was achieved by the following equation [51]:

Ca^2+^ = *K*_d_(F-Fmin)/(Fmax-F) (1)

where *K*_d_ = 400 nmol/L. 

Fmax and Fmin are maximum and minimum fluorescence intensities obtained by Fluo-3/AM calibration after cell exposure to 500 µM A23187 for about 10 min, followed by addition of 20 mM EDTA.

### 4.7. Quantitative Reverse Transcriptase PCR (qRT-PCR) and RNA Interference (siRNA)

Cells were settled in multi-well plates for 24 h and then subjected to the indicated experimental conditions. NucleoSpin RNAII Kit (Macherey-Nagel, Düren, Germany) was utilized to extract total RNA. Complementary DNA was synthesized from RNA using the Transcriptor First Strand cDNA Synthesis Kit (Roche Diagnostics GmbH, Penzberg, Germany).

qRT-PCR was performed using Power Sybr Green Mastermix (Ambion Austin, TX, USA) and KiCqStart® SYBR® Green Primers (Sigma-Aldrich, Table 1) in a CFX384 Real-Time PCR Detection System (Bio-Rad Laboratories, Hercules, CA, USA). Gene expression was measured by the ∆∆*C*t method. 

For NOX-4, RNAi was performed by transfecting cells with 5 µM siRNA oligonucleotides (Sigma-Aldrich, 5’-GAAUGAGUGCAAUUUCUAA-3’ (sense) and 5’-UCCCAUAUGAGUUCUG-3’ (antisense)) or with equimolar scramble siRNA by using the N-ter Nanoparticle siRNA Transfection System (Sigma-Aldrich). 

For TRPM2, RNAi was performed using esiRNA (esiRNA, cat. no. EHU133821, Sigma-Aldrich). Scramble siRNA was obtained using commercial non-targeting siRNA (MISSION siRNA Universal Negative Control). Cells were collected at 24 h after transfection and used for the experiments.

### 4.8. Statistical Analysis

All statistical tests were carried out with GraphPad Prism 5 (GraphPad Software, Inc, San Diego, CA, USA). 

## Figures and Tables

**Table 1:** Sequences of primers used for qRT-PCR.

TARGET GENE	FORWARD SEQUENCE	REVERSE SEQUENCE
β-actin	5’-TCCCTGGAGAAGAGCTACGA-3′	5’-AGCACTGTGTTGGCGTACAG-3′
GADPH	5’-AATCCCATCACCATCTTCCA-3′	5’-TGGACTCCACGACGTACTCA-3′
NOX4	5’-GGTATTGTTCCTCATGGTTAC-3’	5’-TGGGATGATGTCTGGTTAAG-3’
TRPM2	5’-GTGAAGTCATCACTATTGGC-3′	5’-GAATCTCCACACCATATTGC-3′
